# Effect of planned preoperative oral care implemented at least 2 weeks before surgery on postoperative infections: A single-center retrospective observational study

**DOI:** 10.1371/journal.pone.0330165

**Published:** 2025-09-03

**Authors:** Kumiko Furuta, Masashi Hirooka, Yuichi Kotegawa, Yuri Sakamoto, Teruki Miyake, Yumiko Kawamoto, Yoichi Hiasa, Satoshi Hino, Daisuke Uchida

**Affiliations:** 1 Total Medical Support Center, Ehime University Hospital, Toon, Ehime, Japan; 2 Department of Gastroenterology and Metabology, Ehime University Graduate School of Medicine, Toon, Ehime, Japan; 3 Division of Medical Technology, Ehime University Hospital, Toon, Ehime, Japan; 4 Department of Oral and Maxillofacial Surgery, Ehime University Graduate School of Medicine, Toon, Ehime, Japan; Wiltse Memorial Hospital, KOREA, REPUBLIC OF

## Abstract

This study aimed to investigate whether initiating oral care more than 2 weeks before surgery could prevent postoperative infections, particularly pneumonia. This retrospective observational study analyzed 1,806 patients who underwent surgery at the Ehime University Hospital between April 2019 and March 2023. The patients were divided into two groups: those receiving structured oral care at least 2 weeks before surgery (n = 257) and those receiving late or no oral care (n = 1,549). Propensity score matching (PSM) and inverse probability of treatment weighting (IPTW) were used to minimize selection bias. Nevertheless, residual confounding factors, especially confounding by indication, may remain, because as patients who received early oral care may differ systematically from those who did not. The primary outcome measure was the incidence of postoperative pneumonia. After PSM and IPTW analyses, the early oral care group showed significantly lower rates of postoperative pneumonia than the control group (risk difference: −3.56%, 95% confidence interval [CI]: −4.89% to −2.23%, p = 0.0004 in the matched analysis; −3.68%, 95% CI: −4.61% to −2.75%, p < 0.001 in the IPTW analysis). IPTW analysis demonstrated shorter hospital stays in the early oral care than in the control group (mean difference: −2.65 days, 95% CI: −4.75 to −0.55, p = 0.013). Implementing structured oral care at least 2 weeks before surgery reduced postoperative pneumonia and shortened hospital stays across various surgical procedures, suggesting its value as a preventive strategy for improving surgical outcomes.

## Introduction

Advancements in perioperative management and anesthetic techniques have significantly expanded the feasibility of highly invasive surgeries, even in high-risk populations, including older-old patients and those with severe comorbidities [1,2]. However, alongside these advancements, there is an increasing emphasis on reducing surgery-related complications and hospital stays owing to healthcare economic considerations. Among postoperative complications, pneumonia remains a major concern after major surgical procedures, as it is associated with increased mortality and substantial healthcare costs [3].

Oral care is widely practiced in clinical settings, and recent research has highlighted its potential role in reducing systemic infections by controlling endogenous oral infections [4]. Professional oral care interventions have been shown to significantly decrease pathogenic bacteria, including pneumonia-causing bacteria and methicillin-resistant *Staphylococcus aureus* (MRSA), particularly in older patients [5,6]. Despite these findings, current approaches to preoperative oral care have notable limitations. Although studies using propensity score matching (PSM) have demonstrated that perioperative oral care can reduce the risk of postoperative pneumonia —for example, Soutome et al. analyzed 539 patients undergoing esophageal cancer surgery [7] and Ishimaru et al. examined 509,179 patients undergoing various cancer surgeries [8], which are largely confined to specific surgical populations. There is no clear consensus regarding the optimal timing for initiating oral care interventions, which may affect their effectiveness.

A 2-week timeframe has been suggested as the minimum duration necessary for significantly reducing oral pathogens. To ensure sufficient time for infection source elimination (e.g., tooth extraction), oral microbiome control (e.g., controlling bacterial biofilm), and prevention of tooth dislodgement during intubation (e.g., mouthguard fabrication), we believe that at least 2 weeks of preoperative oral care is necessary. This study investigated whether initiating oral care more than 2 weeks before surgery could effectively prevent postoperative pneumonia.

## Methods

### Study design and population

This retrospective observational study included 1,806 patients aged 20 years or older who were admitted to Ehime University Hospital, underwent surgery, and received postoperative management until discharge between April 2019 and March 2023. This study was approved by the relevant institutional ethics committee (IRB No. 2403002) and conducted in accordance with the principles of the Declaration of Helsinki. The data for this study were accessed on April 1, 2024. The authors did not have access to any information that could identify individual participants during or after data collection.

### Patient selection

Patients were selected based on electronic medical record data using the International Classification of Diseases, 10th Revision (ICD-10) diagnostic codes (C codes and I052, I058, I071, I079, I080, I081, I083, I209, I340, I348, I350, I351, I352, I358, I371, I372, Q205, Q211, Q213, and T820) and Diagnosis Procedure Combination (DPC) categories, including stomach, colon, small intestine, peritoneum, esophagus, rectum/anus, head and neck malignancies, lung malignancies, and valvular diseases. These surgical categories were selected because they are associated with a high risk of postoperative pneumonia due to factors such as surgical invasiveness, prolonged operative times, and postoperative impairments in respiratory or swallowing function. Additionally, cancer and cardiovascular diseases may compromise patients’ nutritional and immune statuses, further increasing susceptibility to postoperative infections.

### Data collection

Patient characteristics were collected: sex, age, smoking status, alcohol consumption, body mass index (BMI), performance status (PS), medical history, postoperative hospital stay, diabetes mellitus, anemia, medications, and surgical procedures. The laboratory data included hematological parameters. All data were obtained at the time of hospital admission, prior to surgical intervention, and before the initiation of any oral care procedures in both groups.

### Study groups and outcome measures

Patients were categorized into two groups based on the timing of the oral care intervention. The early intervention group included patients who received structured perioperative oral care for at least 2 weeks before surgery. The late—or non-intervention group included patients who either received oral care within 2 weeks before surgery or did not receive oral care at all.

The primary outcome was the incidence of postoperative infections, including postoperative pneumonia, aspiration pneumonia, surgical site infections, and sepsis, identified using DPC codes for hospital-acquired conditions. The secondary outcomes included postoperative hospital stay and hospitalization costs.

### Oral care protocol

The institutional oral care protocol was implemented at least 2 weeks before surgery in patients with sufficient preoperative time, typically during outpatient visits prior to hospitalization**.** This protocol initially comprised a comprehensive oral evaluation, including dental radiography, periodontal tissue examination, oral hygiene assessment, and identification of potential sources of infection such as dental caries and periodontal disease. To achieve oral bacterial control, dental practitioners treat infected teeth; dental hygienists remove plaque and calculus using ultrasonic scalers and manual instruments; mechanical tooth surface cleaning with fluoride-containing paste; tongue coating removal; and ultrasonic denture cleaning.

Patients received detailed instructions on self-care practices. This instruction encompasses dental plaque and food debris removal from tooth surfaces and interdental areas through appropriate brushing techniques supplemented by auxiliary cleaning devices such as interdental brushes and dental floss. The patients were advised to clean their oral mucosa using tongue and sponge brushes, maintain dentures with specialized cleansing agents, and use chlorhexidine-containing mouthwash rinses.

In patients with insufficient preoperative time, a single session of mechanical tooth surface cleaning was performed. When required, custom mouthpieces were fabricated to prevent tooth displacement during endotracheal intubation. Patients who declined the oral care protocol underwent surgery without implementing these preventive measures.

### Statistical analysis

Statistical analyses were performed using STATA version 18 (StataCorp LLC, College Station, TX, USA). Categorical variables were compared using Fisher’s exact test, and continuous variables were analyzed using Student’s t-test or the Mann–Whitney U test based on their distribution. We employed PSM and inverse probability of treatment weighting (IPTW) to minimize the selection bias.

Propensity scores were calculated using logistic regression with preoperative oral care as the outcome variable and 13 covariates including age, sex, type of surgery, comorbidities (liver cirrhosis, renal failure, diabetes, hypertension, and cardiovascular disease), albumin level, performance status, and BMI. The model’s goodness of fit was assessed using the Pseudo R^2^ value, with values >0.1 considered acceptable. We performed 1:1 matching using the nearest neighbor method with a caliper width of 0.2 of the standard deviation of the logit of the propensity score. To assess the robustness of our findings, a sensitivity analysis was conducted using a strict caliper width of 0.05. The quality of matching was evaluated using standardized mean differences (SMD) and variance ratios, as described above.

The quality of matching was evaluated through several criteria: (1) standardized mean differences (SMD) for all covariates should be < 0.1 after matching, (2) variance ratios of continuous variables should fall within 0.78–1.28, and (3) adequate standard support should be demonstrated in the overlap of propensity score distributions between groups.

For the IPTW analysis, the same propensity scores were used to generate the weights. A balance assessment for IPTW was conducted using the following stringent criteria: (1) SMD for all covariates should be < 0.1; (2) Rubin’s B should be < 25%; and (3) Rubin’s R should be between 0.5 and 2.0. Visual balance assessment was performed using standardized differences and propensity score overlap plots.

The outcome estimates for postoperative pneumonia (primary outcome) and length of hospital stay (secondary outcome) were estimated. The results were presented as risk or mean differences with 95% confidence intervals. Statistical significance was set at p < 0.05. All tests were two-sided.

To ensure the robustness of our findings, we conducted sensitivity analyses by comparing the results of the unadjusted analyses, PSM, and IPTW. The consistency of outcome estimates across these analytical approaches was used to validate our conclusions.

## Results

In total, 1,806 patients were included in this study, with 257 patients in the scheduled oral care group and 1,549 in the control group. Before propensity score matching, patient characteristics were not significantly different in terms of age (median 71 vs. 72 years), sex (male: 67.3% vs. 65.8%), and albumin levels (3.6 vs 3.6 g/dL) (Table 1). However, significant differences were observed in the type of surgery (p < 0.001), with a higher proportion of pulmonary surgeries in the oral care group (159/257, 61.9%) than in the control group (533/1,549, 34.4%). Performance status also differed significantly (PS ≥ 1:30/257, 11.7% vs. 310/1,549, 20.0%, p = 0.0015).

**Table 1:** Baseline characteristics before and after propensity score matching.

	Before propensity score matching			After propensity matching		
Factors	Scheduled oral care group (n = 257) Control group (n = 1549)		p-value	Scheduled oral care group (n = 253) Control group (n = 253)		SMD (%)
**Male**	173 (67.3%)	1,020 (65.8%)	0.646	170: 83	174: 79	1.96
**Age (years)**	71 (65–77)	72 (65–78)	0.086	71 (65–77)	71 (63–76)	7.61
**Type of surgery**			<0.001			5.29
Upper gastrointestinal surgery	25 (9.7%)	284 (18.3%)		25 (9.9%)	36 (14.2%)	
Lower gastrointestinal surgery	24 (9.3%)	318 (20.5%)		24 (9.5%)	27 (10.7%)	
Cardiac surgery	21 (8.2%)	180 (11.6%)		21 (8.3%)	19 (7.5%)	
Pulmonary surgery	159 (62.0%)	533 (34.)		158 (24.5%)	124 (49.0%)	
Head and neck surgery	28 (10.6%)	234 (15.1%)		27 (10.7%)	49 (19.4%)	
**Medical history**						
Liver cirrhosis	1 (0.4%)	8 (0.5%)	0.788	1 (0.4%)	5 (2.0%)	1.57
Renal failure	8 (3.1%)	34 (2.2%)	0.366	7 (2.7%)	5 (2.0%)	0.78
Diabetes	49 (19.1%)	336 (21.7%)	0.341	49 (19.2%)	38 (14.9%)	4.31
Hypertension	77 (30.0%)	448 (28.9%)	0.734	75 (29.4%)	80 (31.4%)	−1.96
Heart disease	49 (19.1%)	368 (23.8%)	0.098	49 (19.2%)	44 (17.3%)	3.30
Respiratory disease	25 (9.7%)	141 (9.1%)	0.748	25 (9.8%)	25 (9.8%)	0.00
Cerebrovascular disease	17 (6.6%)	111 (7.2%)	0.750	17 (6.7%)	14 (5.5%)	1.96
**Albumin (g/dL)**	3.6 (3.2–4.2)	3.6 (3.1–4.1)	0.594	3.6 (3.2–4.2)	3.6 (3.2–4.2)	9.77
**Performance status (0:1≤)**	227:30	1,239:310	0.002	224:29	223:30	1.57
**History of smoking**	162 (63.3%)	921 (60.4%)	0.381	159 (62.9%)	153 (60.5%)	0.00
**Body mass index (kg/m**^**2**^)	22.3 (20.6–25.0)	22.9 (20.5–25.3)	0.298	22.2 (20.6–24.7)	23.1 (20.6–25.2)	1.88

Scheduled oral care group, patients who received preoperative oral care ≥2 weeks before surgery; Control group, patients who received <2 weeks of preoperative oral care or no oral care. Categorical variables are presented as numbers (percentages), and continuous variables are presented as medians (interquartile ranges).

SMD, standard mean difference.

**Fig 1:**
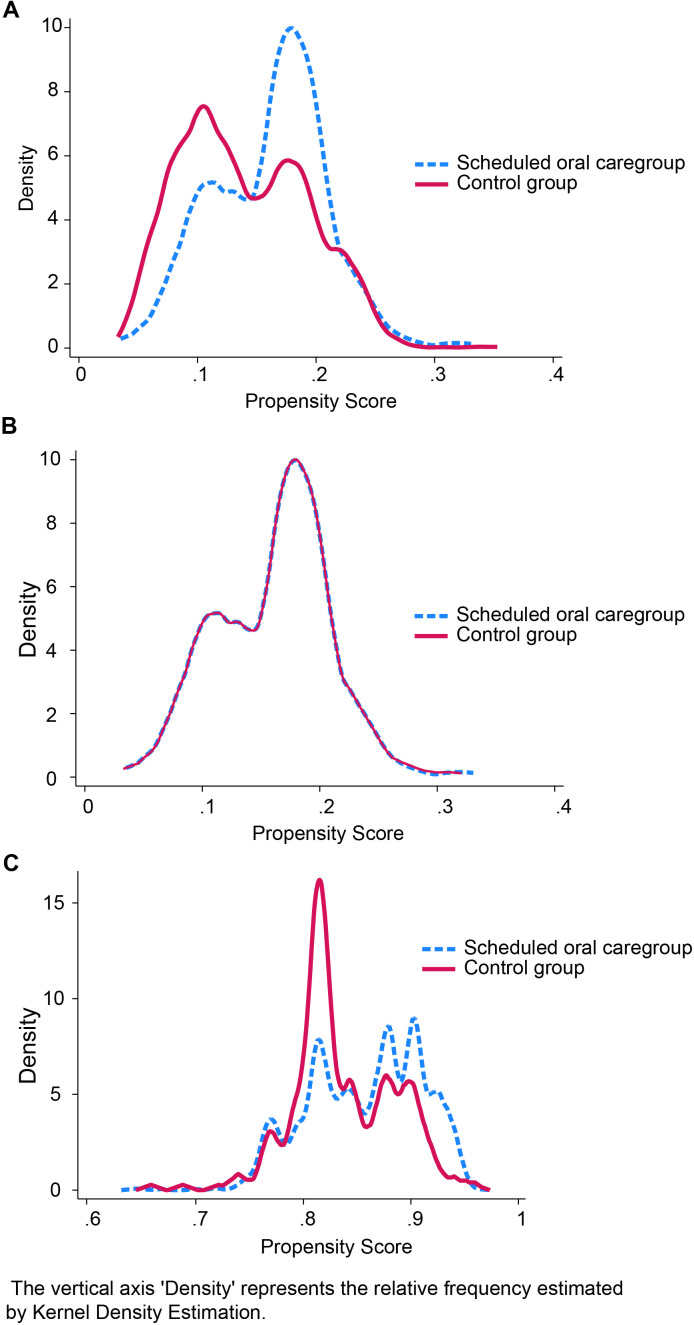
Distribution of propensity scores across treatment groups. **(A)** Distribution before matching shows separation between treated and control groups. **(B)** Distribution after propensity score matching demonstrates improved overlap between matched treated and control groups. **(C)** After the inverse probability of treatment weighting, the distribution shows an optimal balance between groups.

**Fig 2:**
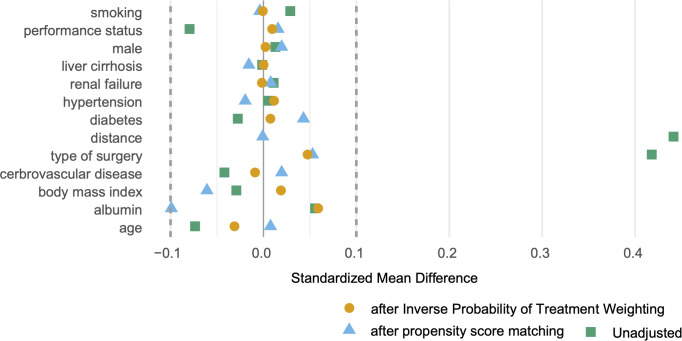
Standardized differences in covariates before and after adjustment. Standardized mean differences (SMDs) for each covariate are displayed, with green squares representing the unadjusted values before matching, blue triangles representing values after propensity score matching (PSM), and orange circles representing values after inverse probability of treatment weighting (IPTW). Dashed vertical lines at ±0.1 indicate the threshold for acceptable covariate balance. After both PSM and IPTW, all covariates achieved SMDs below 0.1, except for albumin, which had a slightly higher SMD of 0.102 after PSM.

The distribution of propensity scores before matching showed some separation between the treatment and control groups, indicating a potential selection bias (Fig 1A). The standardized differences before matching (Fig 2) revealed that three variables exceeded the 10% threshold: type of surgery (37.9%), heart disease (−11.4%), and performance status (−23.0%), indicating a substantial imbalance between groups (Table 2).

**Table 2:** Standardized differences in covariates before and after matching/weighting.

Variables	Before matching	After PSM	After IPTW
Distance	44.14%	−0.07%	not available
Male	1.29%	1.96%	0.22%
Age	−7.36%	0.76%	−3.11%
*Type of surgery	41.80%	5.29%	4.75%
**Medical history**			
Liver cirrhosis	−0.13%	−1.57%	0.01%
Renal failure	1.09%	0.78%	−0.16%
Diabetes	−2.76%	4.31%	0.75%
Hypertension	0.50%	−1.96%	1.14%
Respiratory disease	8.00%	2.00%	0.50%
Cerebrovascular disease	−4.20%	1.96%	−0.90%
Albumin (g/dL)	5.56%	−9.76%	5.89%
Performance status (0:1≤)	−7.95%	1.57%	0.94%
Body mass index (kg/m^2^)	−2.92%	−6.08%	1.88%
History of smoking	2.88%	−0.39%	−0.06%

Values are expressed as percentages (%).

PSM, propensity score matching; IPTW, inverse probability of treatment weighting.

*A less than 10% standardized difference indicates a good balance between groups. Standardized differences in the type of surgery, heart disease, and performance status exceeded 10% before matching but improved to within 10% after PSM and IPTW.

The SMD for distance after IPTW was not calculated due to extreme distributional imbalance following weighting.

After PSM with 14 covariates, 253 matched pairs were identified. The matching process successfully improved the covariate balance, as demonstrated by the overlapping propensity score distributions (Fig 1B) and reduced standardized differences (Fig 2, triangles). After adjustment, all covariates achieved standardized mean differences of < 0.1.

We also conducted an IPTW analysis to check for sensitivity. The propensity score overlap plot after IPTW (Fig 1C) showed excellent group balance. The standardized differences after IPTW (Fig 2, square symbols) were consistently small for all variables (<10%), indicating an optimal covariate balance.

The outcome estimates were evaluated using PSM and IPTW approaches (Table 3). The primary outcome, postoperative pneumonia, significantly reduced both analyses. PSM analysis revealed a significant decrease in pneumonia incidence (risk difference: −5.08%, 95% CI: −8.19% to −1.97%, p = 0.001). IPTW analysis corroborated these findings, showing a similar reduction (risk difference: −3.61%, 95% CI: −4.53% to −2.68%, p < 0.001).

**Table 3:** Outcome estimates after propensity score matching and inverse probability of treatment weighting.

Outcomes	PSM effect (95% CI)	p-value	IPTW effect (95% CI.)	p-value
**Pneumonia incidence (%)**	−5.08 (−8.19 to −1.97)	0.001	−3.61 (−4.53 to −2.68)	< 0.001
**Length of hospital stay (days)**	−2.85 (−6.08 to 0.38)	0.084	−2.55 (−4.66 to −0.45)	0.018
**Medical costs (JPY)**	−5,565 (−13,119 to 1,989)	0.149	−5,385 (−10,445–325)	0.037

The PSM effect shows the average difference between the treated and control groups in a 1:1 matched pair with 95% confidence intervals, indicating the range in which the effect likely lies.

IPTW effect represents the weighted average treatment effect with 95% confidence intervals.

CI, confidence interval; PSM, propensity score matching; IPTW, inverse probability of treatment weighting; JPY, Japanese Yen.

Subgroup analysis by surgery type after IPTW revealed varying incidence rates of postoperative pneumonia across the different surgical procedures (Table 4). The highest incidence was observed in upper gastrointestinal surgery (7.45%, 95% CI: 4.37 to 10.53%), followed by pulmonary surgery (3.92%, 95% CI: 2.27 to 5.58%), cardiac surgery (2.92%, 95% CI: 0.38 to 5.46%), and head and neck surgery (2.54%, 95% CI: 0.51 to 4.58%). Lower gastrointestinal surgery showed the lowest incidence (0.94%, 95% CI: −0.13 to 2.01%).

**Table 4:** Incidence of postoperative pneumonia by surgery type after inverse probability of treatment weighting.

Type of surgery	Control group (%)	Scheduled oral care group (%)
**Upper gastrointestinal surgery**	7.45 (4.37 to 10.53)	0
**Lower gastrointestinal surgery**	0.94 (−0.13 to 2.01)	0
**Cardiac surgery**	2.92 (0.38 to 5.46)	0
**Pulmonary surgery**	3.92 (2.27 to 5.58)	0
**Head and neck surgery**	2.54 (0.51 to 4.58)	0

Notably, no cases of pneumonia were observed in the scheduled oral care group across all surgery types. Values are expressed as percentages with 95% confidence intervals. Since no events were observed after weighting in the scheduled oral care group, confidence intervals could not be estimated.

Secondary outcomes included the length of hospital stay and medical costs. IPTW analysis showed significant reductions in both length of stay (−2.55 days, 95% CI: −4.66 to −0.45, p = 0.018) and medical costs (−5,385 JPY, 95% CI: −10,445 to −325, p = 0.037). While the PSM analysis showed similar directional effects, they did not reach statistical significance (length of stay: −2.85 days, p = 0.084; medical costs: −5,565 JPY, p = 0.149).

### Sensitivity analysis

To assess the robustness of our findings, we conducted a sensitivity analysis using a stricter caliper width of 0.05. The results are summarized in Table 5. Consistent with the primary analysis, the early oral care group demonstrated a significantly lower incidence of postoperative pneumonia than the control group (0% vs. 4.7%, p < 0.001). No significant differences were observed between the groups in terms of postoperative hospital stay (18.62 ± 21.25 vs. 16.16 ± 17.00 days, p = 0.804) or hospitalization costs (48,982 ± 47,533 vs. 44,489 ± 39,751 JPY, p = 0.897).

**Table 5:** Sensitivity analysis using caliper width of 0.05.

Outcome	Control group	Scheduled oral care group	p value
Postoperative pneumonia	12 (4.7%)	0 (0%)	< 0.001
Hospital stays (days)	18.62 ± 21.25	16.16 ± 17.00	0.804
Cost (JPY)	48,982 ± 47,533	44,489 ± 39,751	0.897

Values for categorical variables are presented as number (percentage), and for continuous variables as mean ± standard deviation.

## Discussion

The present study demonstrated that implementing structured oral care at least 2 weeks before surgery significantly reduced postoperative pneumonia, although the baseline risk varied substantially across surgical procedures. This protective effect was robust in PSM and IPTW analyses, with the highest impact observed in upper gastrointestinal surgery, where the risk of pneumonia was the greatest.

The differing results between PSM and IPTW analyses for hospital stay and medical costs require careful interpretation. While PSM analysis showed no significant difference in these secondary outcomes, IPTW analysis demonstrated significant reductions in length of stay (2.55 days) and medical costs (5,385 JPY). These differences may reflect the methodological characteristics of the approaches. PSM analyzes matched pairs, which effectively reduces selection bias but may limit statistical power owing to the reduced sample size. By utilizing an entire cohort, IPTW may better capture the full spectrum of outcome estimates across patient populations [9,10].

The 2-week minimum period for preoperative oral care was supported by microbiological and clinical evidence. From a microbiological perspective, studies have shown that pathogenic oral bacteria require approximately 10–14 days for effective reduction through professional oral care interventions [11–13]. This timeline aligns with the ecological succession principle in oral microbiota, in which the restoration of healthy bacterial flora occurs gradually following a reduction in pathogenic species [14,15]. Professional mechanical cleaning combined with chemical disinfection initiates this microbial shift; however, establishing a stable, healthy oral microbiome requires sustained intervention [16–18].

Technical aspects of professional oral care support this timeline. Initial professional cleaning often reveals areas that require additional attention, and multiple sessions may be necessary to achieve optimal oral hygiene. Patient education and adaptation to new oral care routines also require time to ensure effective implementation [19,20]. From a microbiological and inflammatory perspective, studies have shown that an effective reduction in systemic inflammatory markers through periodontal intervention requires approximately 2 weeks [21]. This finding is supported by a systematic review demonstrating a clear relationship between periodontal infection control and reduction in systemic inflammation markers [22].

We found preoperative oral care effective even in lower gastrointestinal surgeries, where direct anatomical connections to the oral bacteria were less evident. Although there have been few reports on the effects of preoperative oral care in lower gastrointestinal surgery, postoperative pneumonia has been observed in cases of lower gastrointestinal surgery in which planned oral care could not be adequately performed. Similar to other surgical procedures, it is likely that oral bacteria flow into the lungs during intubation in these cases as well. By reducing the bacterial load in the oral cavity through preoperative oral care, the risk of bacterial influx can be decreased in all types of surgery, including upper and lower gastrointestinal procedures.

Our study has several limitations. First, despite the use of robust statistical methods, including PSM and IPTW, unmeasured confounding factors may still exist. Second, although we demonstrated the effectiveness of initiating oral care at least 2 weeks before surgery, we could not determine the optimal duration of preoperative oral care or whether extended periods would provide additional benefits. Third, our study was conducted at a single institution, which may limit the generalizability of our findings to other healthcare settings with different patient populations or surgical practices. While a randomized controlled trial would be the gold standard for establishing causality, it would be ethically challenging to withhold preoperative oral care from control group patients, given the existing evidence of its benefits. Therefore, we plan to conduct a real-world data analysis using a large-scale patient registry that includes multiple institutions to address these limitations. Despite the use of robust statistical methods, residual confounding due to unmeasured variables remains possible. For instance, patients who proactively seek early oral care may generally exhibit better health behaviors, potentially leading to an underestimation of the true effect size. Additionally, confounding by indications may have influenced the results. Patients selected for early oral care might have been perceived by their attending physicians as being at higher risk of postoperative complications, introducing selection bias. Although we attempted to control for measurable confounders using propensity score methods, this potential bias could not be eliminated completely in an observational study.

In conclusion, our findings demonstrate that structured preoperative oral care initiated at least 2 weeks before surgery significantly reduces postoperative pneumonia and shortens hospital stay across various surgical procedures. The consistency of these results across different statistical approaches suggests that implementing standardized preoperative oral care protocols could be a valuable strategy for improving surgical outcomes.
